# Docking of molecules identified in bioactive medicinal plants extracts into the p50 NF-kappaB transcription factor: correlation with inhibition of NF-kappaB/DNA interactions and inhibitory effects on IL-8 gene expression

**DOI:** 10.1186/1472-6807-8-38

**Published:** 2008-09-03

**Authors:** Laura Piccagli, Enrica Fabbri, Monica Borgatti, Valentino Bezzerri, Irene Mancini, Elena Nicolis, Maria C Dechecchi, Ilaria Lampronti, Giulio Cabrini, Roberto Gambari

**Affiliations:** 1 BioPharmaNet, ER-GenTech, Department of Biochemistry and Molecular Biology, University of Ferrara, Italy; 2 Laboratory of Molecular Pathology, Laboratory of Clinical Chemistry and Haematology, University-Hospital, Verona, Italy; 3 Department of Biochemistry and Molecular Biology, University of Ferrara, Italy

## Abstract

**Background:**

The transcription factor NF-kappaB is a very interesting target molecule for the design on anti-tumor, anti-inflammatory and pro-apoptotic drugs. However, the application of the widely-used molecular docking computational method for the virtual screening of chemical libraries on NF-kappaB is not yet reported in literature. Docking studies on a dataset of 27 molecules from extracts of two different medicinal plants to NF-kappaB-p50 were performed with the purpose of developing a docking protocol fit for the target under study.

**Results:**

We enhanced the simple docking procedure by means of a sort of combined target- and ligand-based drug design approach. Advantages of this combination strategy, based on a similarity parameter for the identification of weak binding chemical entities, are illustrated in this work with the discovery of a new lead compound for NF-kappaB. Further biochemical analyses based on EMSA were performed and biological effects were tested on the compound exhibiting the best docking score. All experimental analysis were in fairly good agreement with molecular modeling findings.

**Conclusion:**

The results obtained sustain the concept that the docking performance is predictive of a biochemical activity. In this respect, this paper represents the first example of successfully individuation through molecular docking simulations of a promising lead compound for the inhibition of NF-kappaB-p50 biological activity and modulation of the expression of the NF-kB regulated IL8 gene.

## Background

The main aim of our molecular modelling investigations was to identify natural compounds for their ability to bind to the NF-kappaB p50 as a strategy to identify molecules exhibiting inhibitory activity on the molecular interactions of the transcription factor with its target DNA sequence. p50–p65 heterodimer is the predominant NF-kappaB complex in T-cells regulating HIV-1 infection and recent studies have shown that p50 unit of NF-kappaB is the one that mainly interacts with HIV-1 LTR [1,2]. The specific protein residues involved in DNA binding to the HIV-1 LTR NF-kappaB sites (sequence 5'-GGGACTTTCCC-3') have been identified [3,4]. Structurally different inhibitors of the NF-kappaB/DNA interactions with a rather low binding constant (in the range of 30 μM and 500 μM) are reported in the literature [5-7]. Recently, some molecular modelling studies have predicted possible binding mode of the inhibitors molecules to the DNA binding region of subunit p50, starting from the crystallographic structure of the NF-kappaB homodimer [6-9].

In particular, Sharma et al. [8] in an effort to rationalize the results obtained from EMSA studies on a set of aurintricarboxylic acid analogues, employed docking studies to explain the structure activity relationships observed within this class. To the best of our knowledge, nowadays the identification of new lead compounds for NF-kappaB inhibition through virtual screening of structures libraries is not yet reported in literature. In this paper, we present docking studies on a series of natural compounds previously identified within medicinal plant extracts by us, into NF-kappaB p50 protein target. After evaluation through electrophoretic mobility shift assays (EMSA), we obtained a fairly good agreement between experimental data and molecular modelling identification of bioactive and inactive compounds.

## Methods

### Docking studies

#### Ligands data and preparation

**Figure 1:**
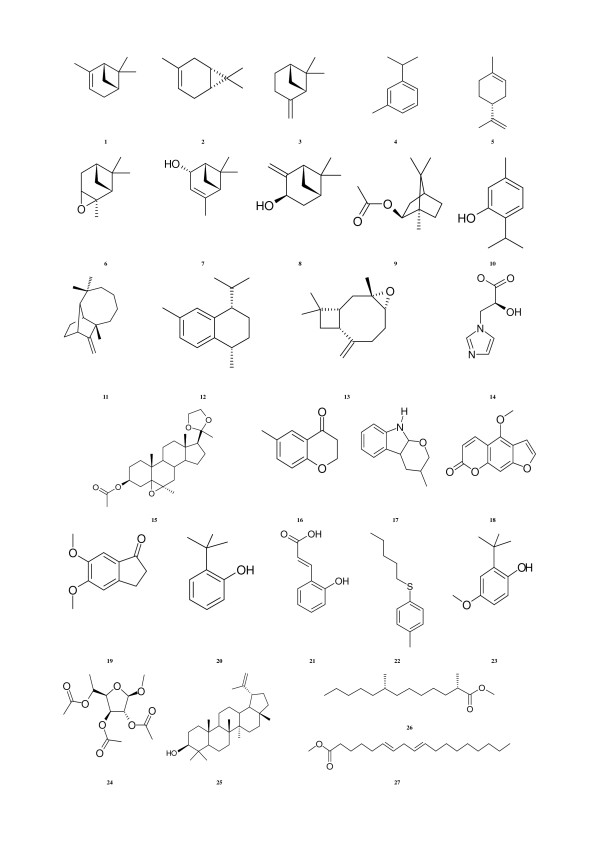
Structures of compounds found in *Cupressus pyramidalis and Aegle marmelos *extracts and used for docking simulations.

**Figure 2:**
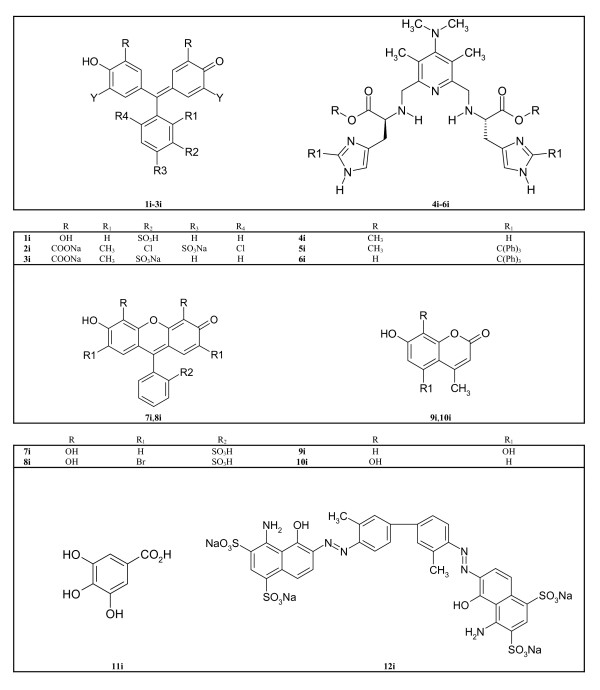
NF-kappaB/DNA binding inhibitors used for atom-pair similarity scoring in docking.

The database of 27 natural structures used in our molecular docking studies, were derived from different medicinal plant extracts (Figure 1) as prepared in our laboratory. A dataset of 12 active compounds used as references molecules were collected from four publications [6-9] reported by one laboratory (Figure 2). Ten of these inhibitors (**1i-8i**, **11i **and **12i**) were employed in starting docking studies (protocol 1) and in the Standard Similarity Scoring for subsequently docking simulations.

Two inhibitory molecules (**9i **and **10i**) were used as test set in all docking simulations. The three-dimensional models of all the molecules under investigation were built by assembling fragments from the SYBYL 7.0 software package standard library [10]. Resulting geometries were optimized and molecular charges were assigned by a semi empirical molecular orbital calculations using the AM1 Hamiltonian [11] (module MOPAC implemented in SYBYL).

#### Proteins data and preparation

The three dimensional structure of the complex NF-kappaB-DNA [4] was retrieved from the Protein Data Bank (PDB code: 1NFK). The cocrystallized DNA macromolecule was removed from the structure. p50 dimer and p50 monomers (chains A and B) were selected for the docking simulations and prepared using the graphical interface Maestro [12]. All water molecules were removed, the hydrogen atoms were added to the proteins and all atom force field (OPSL-2001) charges and atom types were assigned. Preparation and refinement were done running ProteinPrep job on the structure in a standard procedure. Minimizations were performed until the average root mean square deviation of non-hydrogen atoms reached 0.3 Å.

#### Docking Simulations

All molecules of plant extracts (**1–27**) and the known inhibitors (**1i-12i**) under study were docked in to the binding site of the receptor (PDB ID: 1NFK) using Glide (Grid-Based Ligand Docking With Energetics) software from Schrodinger [13,14]. Grids were prepared for each proteins with the exact same center and the size of the bounding box set on 30 Å. The coordinates of the enclosing box (x = -1,1958 Å; y = 9.0149 Å; z = 19,7598 Å) were defined starting from the set of active site residues involved in hydrogen bonds with the NF-kappaB recognition site of DNA (Arg54, Arg56, Tyr57, Cys59, Lys241, Gln306 and Thr143) and optimised including the double strands DNA helices volume by visual inspection. The Glide algorithm is based on a systematic search of positions, orientations, and conformations of the ligand in the receptor binding site using funnel type approach. The search begins with a rough positioning and scoring phase that significantly limits the search space and reduces the number of poses to be selected for minimization on the precomputed OPLS-2001 van der Waals and electrostatic grids for the protein. The 5–10 lowest-energy poses obtained from this stage are subjected to Monte Carlo simulations and the minimized poses accepted are then rescored using the GlideScore function, which is a more sophisticated version of ChemScore [15]. This force field include additional terms accounting for solvation and repulsive interactions. In order to provide a better correlation between good poses and good scores, Glide Extra-Precision (XP) Mode was subsequently used on the conformations selected from Glide Standard Precision (SP) mode. The atom-pair superimposition of p50 chains A and B, prepared as described above, gave a minimum RMSD of 2,303 Å (heavy atoms). Considering the clear dependence of the docking accuracy of ligands on the protein structure, docking simulations were carried out with the same protocol on both A and B, considered as two slightly different conformations of the same structure.

Unfortunately, complexes of NF-kappaB cocrystallized with inhibitors has not been solved. Therefore, a common self-docking procedure to evaluate the accuracy of the docking protocol adopted was not practicable. In order to overcame this situation, two structurally similar active compounds (**9i **and **10i**) were used as test set and docked into the DNA binding site of the protein. Moreover in following docking jobs, atom pair similarity (AP) scoring (Similscore) facility as implemented in Glide, was incorporated in GlideScore (G-score), based on the assumption that closely related chemical structure should share similar biological activity and physiochemical property [16]. Similscore can have a value between 0 and 1 as implemented in Glide. The adjusting G-score value is illustrated here below:

1. if 0.0 ≤ SimilScore < 0.3 → G-score = G-score+6.0

2. if 0.3 ≤ SimilScore < 0.7 → G-score = G-score+(0.7-Similscore)/(0.7-0.3)*6.0

3. if 0.7 ≤ SimilScore < 1.0 → G-score = G-score+0.0

All inhibitors molecules, except for **9i **and **10i**, were used just as reference structures for AP similarity method.

Based on the best final GlideScore ranking, the similarity docking procedure for subsequently docking simulations on p50 subunits was chosen.

### Preparation of nuclear extracts

Nuclear extracts were prepared as described [18]. Cell were washed twice with PBS and detached by trypsinization. After homogenization with Dounce B homogeneizer, nuclear proteins were obtained and protein concentration was determined using Bio-Rad protein assay. Nuclear extracts were brought to a concentration of 0.5 μg/μl for Electrophoretic Mobility Shift Assay (EMSA) experiments [18].

### Electrophoretic Mobility Shift Assay (EMSA)

EMSA was perfomed as previously described [18-20]. Briefly, double-stranded synthetic oligodeoxynucleotides mimicking the NF-κB binding site present in the promoter of the IL-8 gene (IL-8 NF-κB, sense: 5'-AAT CGT GGA ATT TCC TCT-3') have been employed. Oligodeoxynucleotides were labeled with γ^32^-P-ATP using 10 Units of T4-polynucleotide-kinase (MBI Fermentas) in 500 mM Tris-HCl, pH 7.6, 100 mM MgCl_2_, 50 mM DTT, 1 mM spermidine, 1 mM EDTA in the presence of 50 μCi γ^32^-P-ATP) in a volume of 20 μl for 45 minutes at 37°C. Reaction was brought to 150 mM NaCl and 150 ng complementary oligodeoxynucleotide was added. Reaction temperature was increased to 100°C for 5 minutes and left diminishing to room temperature overnight. Nuclear extracts from IB3-1 cells or purified NF-κB p50 dimer protein (Promega) were used at the specified concentrations and poly(dI:dC) (1 mg per reaction) was also added to abolish nonspecific binding [21]. After 5 min binding at room temperature, the samples were run at constant voltage (200 V) under low ionic strength conditions (0.25× TBE buffer: 22 mM Tris-borate, 0.4 mM EDTA) on 6% polyacrylamide gels. Gels were dried and subjected to standard autoradiographic procedures.

### Cell cultures and infection with *Pseudomonas aeruginosa*

IB3-1 cells have been obtained from LGC Promochem [20]. Cells have been grown in LHC-8 basal medium (Biofluids), supplemented with 5% FBS in the absence of gentamycin [21]. All culture flasks and plates have been coated with a solution containing 35 g/ml bovine collagen (Becton-Dickinson), 1 g/ml bovine serum albumin (Sigma) and 1 g/ml human fibronectin (Becton-Dickinson) as described. P. aeruginosa, PAO1 strain, was grown in trypticase soy broth (TSB) or agar (TSA) (Difco). Bacteria colonies from o/n cultures on TSA plates were grown in 20 ml TSB at 37°C. IB3-1 cells were infected with ranging doses of PAO1 at 37°C for 4 hours.

### Quantitation of transcripts of inflammatory genes

Total RNA from IB3-1 cells was isolated using High Pure RNA Isolation Kit (Roche. Mannheim. Germany) [21]. Total RNA (2.5 μg) was reverse-transcribed to cDNA using the High Capacity cDNA Archive Kit and random primers (Applied Biosystems) in a 100-μl reaction. The cDNA (2 μl) was then amplified for 50 PCR cycles using the Platinum^® ^SYBR^® ^Green qPCR SuperMix-UDG (Invitrogen) in an ABI Prism 5700 sequence detection system (Applied Biosystems). The real-time PCR reactions were performed in duplicates for both target and normalizer genes. Primer sequences for detection of IL-8 mRNA were GACCACACTGCGCCAACA (IL-8 forward) and GCTCTCTTCCATCAGAAAGTTACATAATTT(IL-8 reverse). Primer sets were purchased from Sigma-Genosys (The Woodlands. TX). Results were collected with Sequence Detection Software (version 1.3; Applied Biosystems). Relative quantification of gene expression was performed using the comparative threshold (C_T_) method as described by the manufacturer (Applied Biosystems User Bulletin 2). Changes in mRNA expression level were calculated following normalization to calibrator gene [22]. The ratios obtained following normalization are expressed as -fold change over untreated samples.

## Results and discussion

### Docking analysis

The docking results for all the reference inhibitory compounds and the natural compounds under study are reported in Table 1 (references compounds) and in Table 2 (natural compounds). As shown in the 2.3 Å crystal structure, the DNA/p50 complex is formed by one DNA molecule and two p50 proteins each one consisting of two distinct domains connected by a10-residue linker. Both domains and the segments that connects them, form a sequence-specific DNA-binding surface by contributing 5 loops per subunit that fill the entire major groove of the DNA. The specific interactions that stabilized the NF-kappaB/DNA complex, occur over 10-bp forming the kB recognition site. Unlike many dimeric protein-DNA complexes, many residues of both subunits make specific base contacts in a non-contiguous cooperative network. The plasticity of centre region of the interface carry to the lack of symmetry exhibited by the interactions of Lys 241 from the linker segment, and Lys 272 and Arg 305 from the dimerization region with the symmetrical target site [4]. In the subsequent experimental EMSA studies, a recombinant p50 protein that probably forms a monomer-dimer mixture in binding buffer solution will be used. On the base of structural and experimental assumptions as above mentioned, p50 dimer and monomer were employed as protein target in our molecular modelling investigation.

**Table 1:** Ranking of the poses of references inhibitory molecules (1i-8i and 11i-12i) in the target NF-kappaB p50 both as dimer (p50-p50) and as monomers (p50 A and B).

**Docking protocol 1**					
***p50-p50***		***p50 (A)***		***p50 (B)***	
		
**Cpd.**	**G-Score**	**Cpd.**	**G-Score**	**Cpd**	**G-Score**

**8i**	-5.88	**8i**	-6.06	**1i**	-6.08
**7i**	-5.88	**7i**	-5.83	**3i**	-5.35
**2i**	-5.84	**11i**	-5.20	**7i**	-5.06
**3i**	-5.78	**1i**	-5.07	**2i**	-4.58
**11i**	-3.87	**2i**	-2.79	**11i**	-3.59
**1i**	-3.57	**3i**	-2.77	**4i**	-1.82
**4i**	-0.76	**4i**	> 0	**8i**	> 0
**12i**	> 0	**12i**	> 0	**12i**	> 0
**5i**	-	**5i**	> 0	**5i**	-
**6i**	-	**6i**	> 0	**6i**	-

**Table 2:** Ranking of the poses of natural compounds and test set inhibitors (9i and 10i) in the target NF-kappaB p50 both as dimer (p50-p50) and as monomers (p50 A and B). In the docking protocol 1 the similarity scoring algorithm is not used.

**Docking protocol 1**		**Docking protocol 2**					
	
***p50-p50***		***p50-p50***		***p50 (A)***		***p50 (B)***	
			
**Cpd.**	**G-score**		**G-Score**	**Cpd.**	**G-Score**	**Cpd.**	**G-Score**
***10i***	*-5.61*	***21***	*-2.34*	***9i***	*-3.81*	***9i***	*-2.13*
**15**	-5.50	***9i***	*-2.29*	***10i***	*-2.27*	***10i***	*-1.43*
**25**	-5.24	***10i***	*-2.19*	***21***	*-1.40*	***21***	*-0.32*
**23**	-4.94	**18**	-0.50	**18**	-0.07	**18**	> 0
***21***	*-4.91*	**23**	-0.05	**10**	> 0	**15**	> 0
**24**	-4.74	**20**	> 0	**23**	> 0	**23**	> 0
***9i***	*-4.44*	**15**	> 0	**7**	> 0	**24**	> 0
**10**	-4.40	**25**	> 0	**8**	> 0	**10**	> 0
**20**	-4.37	**24**	> 0	**20**	> 0	**23**	> 0
**7**	-4.28	**10**	> 0	**15**	> 0	**20**	> 0
**22**	-4.13	**16**	> 0	**25**	> 0	**16**	> 0
**16**	-3.48	**7**	> 0	**24**	> 0	**7**	> 0
**19**	-3.71	**22**	> 0	**13**	> 0	**8**	> 0
**2**	-3.39	**19**	> 0	**12**	> 0	**2**	> 0
**27**	-3.09	**4**	> 0	**16**	> 0	**22**	> 0
**5**	-3.05	**2**	> 0	**6**	> 0	**4**	> 0
**26**	-2.89	**5**	> 0	**9**	> 0	**1**	> 0
**18**	-2.69	**27**	> 0	**11**	> 0	**19**	> 0
**14**	-2.47	**26**	> 0	**2**	> 0	**14**	> 0
**4**	-2.00	**14**	> 0	**3**	> 0	**5**	> 0

In order to evaluate the impact of the introduction of the similarity penalty in the docking algorithm on the results, the positions of **9i **and **10i **used as test set in the final GlideScore ranking were compared in the two different procedures (Table 2).

Known active compound **10i **was ranked at the top positions in both procedures, but only the introduction of the similarity parameter in the scoring function significantly increased the efficiency in **9i **ranking (Table 2). In fact the difference in glide-score among these two inhibitors with a similar inhibitory potency (500 μM) [9] was very small (ΔGlideScore = 0.10). For each selected ligand the pose with best E-Model score (combination of energy grid score, GlideScore, and the internal strain of the ligand) was used for in-deph interaction analysis. Compound **21 **clearly showed highest score in respect to docked plant extracts (Table 2) outranking the known inhibitors at physiological pH in docking simulation to the dimer.

**Figure 3:**
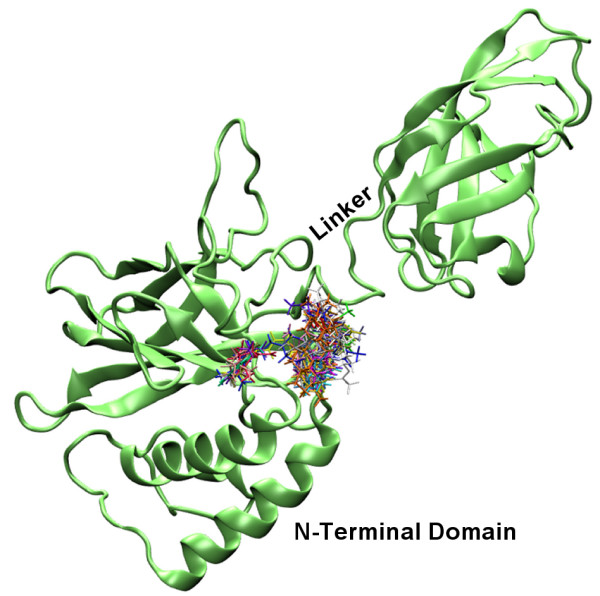
Stereoview of compounds **1–27**, **9i **and **10i **docked in to DNA binding region of the NF-kappaB p50 monomer chain A. The macromolecule is highlighted in green.

**Figure 4:**
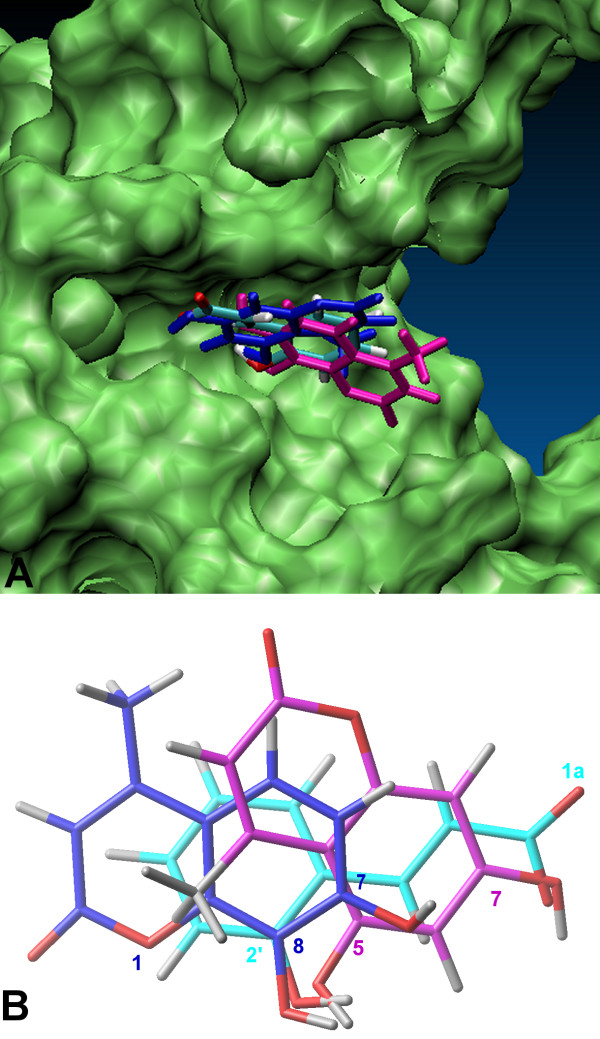
Superimposition of the docked poses of inhibitors **9i**, **10i **and compound **21**. A. the DNA binding site of NF-kappaB p50 (monomer chain A) is highlighted in green; B. the ligand atoms involved in hydrogen bonding are labeled. Compounds **9i**, (shown in purple), **10i **(shown in blue) and **21 **are illustrated in stick representations.

Docked compounds **1–27**, **9i **and **10i**, occupied a region of the binding surface creates by the spatial relationship between the N-terminal domain of p50 subunit and the 10 residues long linker loop (Figure 3). Molecules **21**, **9i **and **10i **(Figure 4) were located in a small cleft surrounded by several polar amino acids (i.e. Tyr57, His109, His141, Tyr143 Lys144, Lys145, Ser208, Asp239, Lys241 and Ser208) and the highest score poses were superimposable with minimum RMSD of 1.36 Å for compounds **9i **and **21**. The RMSD was calculated by superimposing the following atoms pairs: heteroatoms involved in hydrogen bonding with the same residues of the protein (**9i**.O8 and **21**.O2'; **9i**.O7 and **21**.O1a) (Figure 4B) and the centroid of aromatic system of coumarin structure with the centroid of benzene ring of **21**. These compounds showed slightly different binding modes in p50 (chain A), p50 (chain B) and p50-p50 targets. Here we reported the highest score poses obtained from docking protocol including the similarity function. H-bond interactions between OH groups of coumarin structures (OH of benzene ring in **21**) and both NH of His141 and the carboxylic group of Asp239 showed to be important for ligands binding.

**Figure 5:**
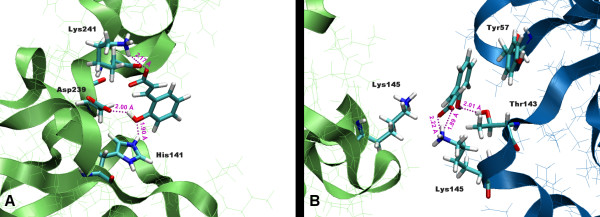
Binding modes of **21 **docked in to the active site of NF-kappaB p50: A. monomer and B. homodimer (chain A, shown in green). The residues involved in the interaction with the ligand are shown; the hydrogen bonding and the relative distances are indicated in purple.

**Figure 6:**
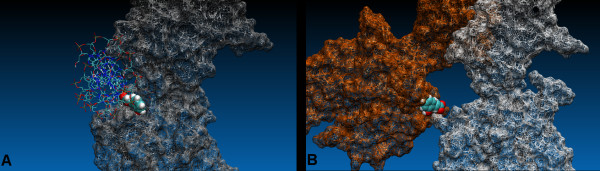
Poses of docked compound **21 **in to the DNA binding region of NF-kappaB p50. A. monomer and B. homodimer (chain A, shown in grey). The inhibitory activity of **21 **may be due to its ability to form a stable complex with the active conformation of the dimer and/or blocking the interaction of DNA with the monomer filling the protein binding site. The DNA was obtained from the crystal structure of the homodimer NF-kappaB (pdb code:1NFK). The surface of the protein is represented in wire frame, the ligand and DNA are highlighted in VDW and stick representation respectively.

Moreover, OH groups of coumarin moiety (carboxylate group in **21**) made an additional hydrogen bond with CO of the backbone and protonated NH_3 _group of Lys241 (Table 3). It is important to note that Lys241 could be involved in the stability of the DNA-binding conformation of the protein. In fact, as discussed above, this residue is situated in the flexible linker segment and interacts with Lys 272 and Arg 305 from the dimerization domain. Finally, carbonyl group of **10i **engage another H-bond with NH of the backbone of the Leu207. Compound **21 **showed the same binding mode of active ligands in the monomer configuration of the target, with the only difference of a stronger interaction of carboxylate group with Lys241 (Table 3 and Figures 5A, 6A).

**Table 3:** intramolecular hydrogen bonds of the docked poses of 9i, 10i and 21 with the involved residues of the DNA binding region of NF-kappaB (see Figure 5 for ligand atom labels). The interatomic distances in Angstroms are shown.

Residue interaction	Ligand atom	Distance (Å)
**(His141) **-Nε-H::O	O5 **(9i)**	2.12
	O8 **(10i)**	1.88
	O2' **(21)**	1.90

**(Asp239) **-COO::H	H5 **(9i)**	1.91
	H8 **(10i)**	2.09
	H2' **(21)**	2.00

**(Lys241) **-CO::H	H7 **(10i)**	1.96

**(Lys241) **-N^+^-H::O	O7 **(9i)**	2.60
	O1a **(21)**	2.17

**(Leu207) **-N-H::O	O1 **(10i)**	2.18

Interestingly, the best pose of compound **21 **occupied a region formed by residues of both p50 units (chain A and chain B) of NF-kappaB dimer: Lys 145 and Thr143 of chain A and Tyr57, Lys144, Lys145, Glu60, Cys59, Thr143, Lys146 of chain B. In particular, the OH group of the ligand engages a hydrogen bond with the sidechain of Thr143 (chain B), and the carboxylate group forms a salt bridge stabilized by two hydrogen bonds with the side chain of Lys 145 (chain B). Moreover the phenyl structure of compound **21 **could be involved in a weak π-π stacking interaction with the aromatic moiety of Tyr57 (chain B) (centroid-centroid distance: 4.93 Å), a residue specific for kB DNA sequence 5'-GGGATTTCC-3', present in different cellular genes including HIV-LTR. Of course, further dynamics simulation on the protein-ligand complex should be necessary to validate this hypothesis. In addition, the amino group of Lys145 of the opposite p50 unit (chain A) could form an additional π-cation interaction with the aromatic group of **21 **(N^+^-H and centroid of benzene ring distance: 3.87 Å) (Figures 5B, 6B). These bridge structures are likely to reinforce the anchoring of this molecule to the DNA binding region of the dimer, and might account for the slight better G-score of **21 **in respect to the monomer configuration of the receptor. Moreover, all the residues of the protein involved in molecular interactions with molecule **21**, form hydrogen bonds also with DNA.

All compounds with higher GlideScore and E-Model score clearly showed the ability to make a maximum number of hydrogen bonding, according with the result as previously reported on a flexible docking studies of known inhibitors **9i **and **10i **[9], even if reported residues involved in binding interaction were different. The highest ranking poses of **21**, **9i **and **10i **formed 3–4 hydrogen bonding with the target protein, whereas molecules in medium positions in docking ranking not more than 2. According, structures not involved in hydrogen bonding were ranked in the last positions (Table 2). In particular, compound **5 **with a GlideScore < 0 in similarity protocol lost the ability both to occupy the same positions of active ligands and to form hydrogen bonding with the protein (not shown). In house experimental data were in good agreement with the molecular modelling findings. In accordance with docking results, **21 **and **5 **showed to be active and inactive respectively in further EMSA experimental studies.

### Effects of compound 21 on NF-kappaB/DNA interactions

**Figure 7:**
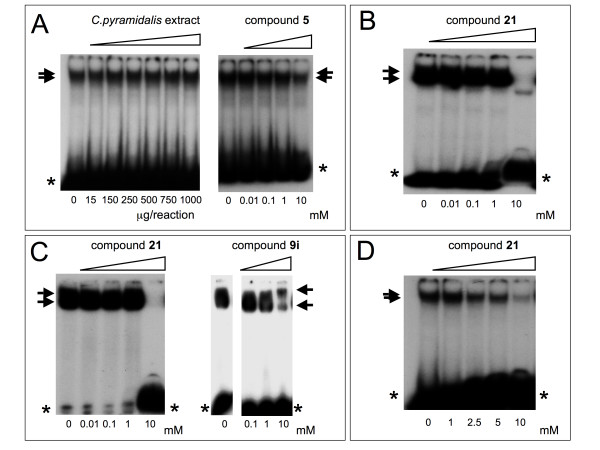
EMSA analysis. NF-kappaB p50 has been incubated for 15 min in the presence of increasing amounts of compounds **5 **(right side of panel A), extracts from *C. pyramidalis *(left side of panel A), compound **21 **(B-D) and reference compound **9i **(right side of panel C). After the incubation of compounds (or *C. pyramidalis *extracts) to NF-kappaB p50, a further 15 min incubation step was carried on with a ^32^P-labelled double stranded oligonucleotide carrying the NF-kappaB binding sites. *C. pyramidalis *extracts were employed at a concentration ranging from 15 to 1000 μg/reaction as mentioned. Comparison between compound **21**, compound **5**, and compound **9i **was performed at 0.01, 0.1, 1 an 10 mM concentrations. Concentration-dependency of the effects displayed by compound **21 **was further analysed with intermediate dosages in the experiment depicted in panel D. The NF-kappaB/DNA complex were identified after polyacrylamide gel electrophoresis. Arrows indicate NF-kappaB/DNA complexes. Asterisks indicate free target DNA.

The effects of compound **21 **on NF-kappaB interactions were first studied by electrophoretic mobility shift assay (EMSA) as described elsewhere [18-21]. It is indeed well accepted that molecules binding NF-kappaB might retain inhibitory activity on molecular interaction between NF-kappaB and DNA [21]. Accordingly, we performed EMSA in the presence of increasing amounts of compound **21**. In addition, compounds **5 **was used as possible negative control. This compound, indeed, is expected, from the docking analysis (Table 2), to be less active. In addition, extracts from *Cupressus pyramidalis *were also used, since this extract does not inhibit NF-kappaB/DNA interactions (data not shown). Finally, the known inhibitory compound **9i **was used as reference molecule. The results of the gel retardation analysis are shown in Figure 7 and clearly demonstrate that compound **21 **inhibit the molecular interactions between nuclear factors (Figure 7B) or isolated NF-kappaB p50 (Figure 7C and 7D) and a target double stranded oligonucleotide mimicking the NF-kappaB binding sites. This effect was similar to that exhibited by the reference compound **9i**. Interestingly, compound **5 **and extracts from *C. pyramidalis *were found to be inactive (Figure 7A), fully in agreement with the docking data summarized in Table 2.

### Biological effects of compound 21: inhibition of *Pseudomonas aeruginosa *mediated increase of IL-8 mRNA

Several experimental model system are available for biological validation of molecules inhibiting NF-kappaB function. In a recent paper we report that decoy oligonucleotides targeting NF-kappaB are powerful inhibitors of *Pseudomoas aeruginosa *mediated induction of IL-8 in cystic fibrosis IB3-1 cells [21]. Besides the importance of these data for the theoretical point of view, our results are of great interest for the practical point of view, suggesting this treatment as a possible strategy for the therapy of inflammation associated with cystic fibrosis.

**Figure 8:**
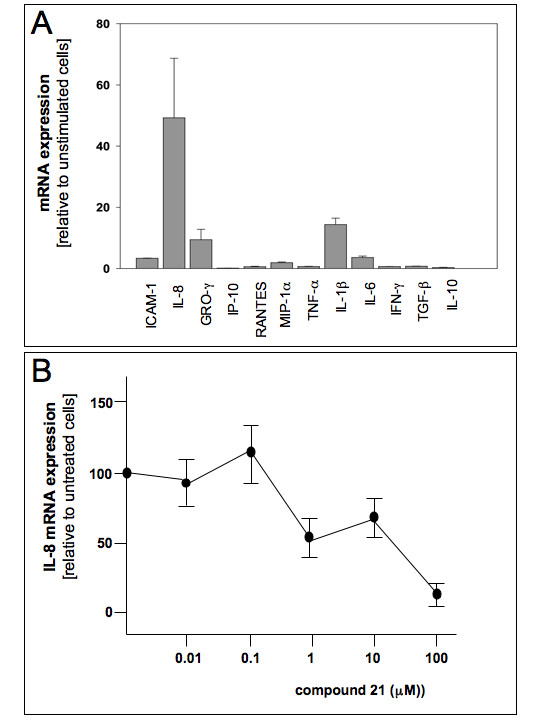
A. Effects of PAO-1 infection of cystic fibrosis IB3-1 cells on the expression of the indicated mRNA. Cells were infected with PAO-1 for 4 hours and then the mRNA analysed by RT-PCR. For RT-PCR analysis the PCR primers have been described in Bezzerri et al. [21] B. Effects of compound 21 on the accumulation of IL-8 mRNA. Cells were incubated for 24 hours in the presence of the indicated concentrations of compound 21, then infected with 150 cfu/cell of PAO-1. Accumulation of IL-8 mRNA was determined by RT-PCR analysis [21].

When the effects of *P. aeruginosa *infection on the expression of pro-inflammatory genes of IB3-1 cells infected for 4 hours are analysed, the results shown in Figure 8A are obtained. In this preliminary experiment the content of RNAs coding for several pro-inflammatory proteins was analysed by RT-PCR. The results obtained indicate that IL-8 mRNA sequences sharply increases following PAO1 infection by 40 folds (range 12–92) in respect to basal level of uninfected cells, assumed to be 1. In addition to IL-8 mRNA, other genes induced by PAO1 are GRO-γ (15 folds, range 9–36), IL-6 (18 folds, range 5–44), IL-1β (5 folds, range 2–8), ICAM-1 (4 folds, range 2–8). On the contrary, very low increase of accumulation of IP-10, RANTES, MIP-1α, TNF-α, IFN-β, TGF-β and IL-10 mRNA was observed under these experimental conditions.

Since NF-kappaB is one of the most important transcription factors regulating the expression of IL-8 gene [23] and the data reported in Figure 7 demonstrate that compound **21 **inhibit NF-kappaB/DNA interactions, we tested the activity of this compound in inhibiting the expression of IL-8 gene in IB3-1 cells infected with PAO1. Cells were exposed to different concentrations of compound **21 **and then infected with 150 cfu/cell of PAO1. After 4 hours, cells were harvested, RNA extracted and quantitative RT-PCR analysis performed. The results obtained (Figure 8B) demonstrate that compound **21 **is a strong inhibitor of PAO-1 induced accumulation of IL-8 mRNA.

## Conclusion

In the present work, we carried out docking studies on the dataset of 27 molecules found in different plant extracts to NF-kappaB-p50, with the purpose of developing a docking protocol fit for the target under study, eventually applicable for more time-consuming virtual screening of larger database of compounds.

Usually, docking to protein structures that do not have a ligand present, as in the case of NF-kappaB, dramatically reduces the expected performance of structure-based methods. Therefore, the use of NF-kappaB as a target for virtual docking of natural compounds is not feasible. To overcome such a limitation, we proposed to enhance the simple docking procedure by means of a sort of combined target- and ligand-based drug design approach. Advantages of this combination strategy, based on a similarity parameter for the identification of weak binding chemical entities, are illustrated in this work with the discovery of a new lead compound for NF-kappaB. In this respect, this paper represents the first example of successfully individuation of a potential lead compound through molecular docking simulations on a NF-kappaB target. At the same time, information derived from this structure and its different binding modes, could carry through further lead optimization to more potent NF-kappaB inhibitors.

In order to validate the approach, biochemical analyses based on EMSA were performed on compound **21**; the results obtained sustain the concept that the docking performance is predictive of a biochemical activity (Figure 7).

Our results are of interest also from the practical point of view. The transcription factor NF-kappaB is indeed a very interesting target molecules in the design on anti-tumor, anti-inflammatory, pro-apoptotic drugs.

In order to validate this last hypothesis, we have employed human cystic fibrosis IB3-1 tracheal epithelial cells. We have elsewhere reported that these cells activate, upon exposure to the bacterium *Pseudomonas aeruginosa *(the PAO-1 strain), the expression of several pro-inflammatory genes, including those coding interleukin-6 (IL-6) and interleukin-8 (IL-8). As supported by several groups, the expression of IL-8 is dependent from NF-kappaB activation. Accordingly, decoy molecules targeting NF-kappaB are strong inhibitors of the IL-8 expression. Therefore, PAO-1 infected IB3-1 cells are a very interesting model system to screen for IL-8 inhibitors. The results of our experiments, in agreement with both docking and EMSA data, demonstrate that compound **21 **is a strong inhibitor of IL-8 and should be considered of interest for modulation of the expression of this gene.

## Authors' contributions

LP carried out all the bioinformatic procedures and the docking experiments. EF participated to the EMSA assays; MB purified the nuclear factors for EMSA analysis; I.M. performed semi-quantitative RT-PCR analysis; VB performed the treatment of IB3-1 cells with selected compounds; MCD performed infection IB3-1 cells with P. aeruginosa; EN performed quantitative RT-PCR analysis of IB-8 mRNA; GC was the responsible of the conception, design, analysis and interpretation of the data on cystic fibrosis cell lines; RG was the responsible of the coordination of the project and of the drafting of the manuscript. All authors read and approved the final manuscript.
